# Salvage Surgery with Intraoperative Electron Radiation Therapy for Recurrent or Persistent Head and Neck Cancer After Prior Radiotherapy

**DOI:** 10.3390/cancers18203399

**Published:** 2026-10-21

**Authors:** Ahmed N. Elguindy, Sujith Baliga, Brett G. Klamer, Allison Palmiero, Khaled N. Dibs, Andrew Koempel, Emile Gogineni, David Konieczkowski, Darrion L. Mitchell, Nolan Seim, Kyle VanKoevering, Catherine Haring, Lauren Miller, Stephen Y. Kang, Amit Agrawal, Enver Ozer, James W. Rocco, Ricardo L. Carrau, Matthew Old, Marcelo Bonomi, Priyanka Bhateja, Arnab Chakravarti, John Grecula, Dukagjin M. Blakaj

**Affiliations:** 1 Department of Radiation Oncology, The James Cancer Hospital/Wexner Medical Center, The Ohio State University, Columbus, OH 43210, USA; ahmed.elguindy@osumc.edu (A.N.E.); sujith.baliga@emory.edu (S.B.); allison.palmiero@osumc.edu (A.P.); khaled.dibs2@osumc.edu (K.N.D.); andrew.koempel@rockets.utoledo.edu (A.K.); emile.gogineni@osumc.edu (E.G.); david.konieczkowski@osumc.edu (D.K.); darrion.mitchell@osumc.edu (D.L.M.); arnab.chakravarti@osumc.edu (A.C.); john.grecula@osumc.edu (J.G.); 2 Department of Radiation Oncology, El-Demerdash Hospitals, Ain Shams University, Cairo 11566, Egypt; 3 Department of Radiation Oncology, Winship Cancer Institute of Emory University, Emory University School of Medicine, Atlanta, GA 30322, USA; 4 Center for Biostatistics, College of Medicine, The Ohio State University, Columbus, OH 43210, USA; brett.klamer@osumc.edu; 5 Department of Otolaryngology-Head and Neck Surgery, The James Cancer Hospital/Wexner Medical Center, The Ohio State University, Columbus, OH 43210, USA; nolan.seim@osumc.edu (N.S.); kyle.vankoevering@osumc.edu (K.V.); catherine.haring@osumc.edu (C.H.); lauren.miller@osumc.edu (L.M.); stephen.kang@osumc.edu (S.Y.K.); amit.agrawal@osumc.edu (A.A.); enver.ozer@osumc.edu (E.O.); james.rocco@osumc.edu (J.W.R.); ricardo.carrau@osumc.edu (R.L.C.); 6 Department of Otolaryngology-Head and Neck Surgery, University of Washington, Seattle, WA 98195, USA; mattold@uw.edu; 7 Department of Medical Oncology, The James Cancer Hospital/Wexner Medical Center, The Ohio State University, Columbus, OH 43210, USA; marcelo.bonomi@osumc.edu (M.B.); priyanka.bhateja@osumc.edu (P.B.); 8 Adjunct Faculty of Medicine, University of Prishtina, St. George Bush, No. 31, 10000 Prishtina, Kosovo

**Keywords:** head and neck cancer, recurrent cancer, salvage surgery, intraoperative electron radiation therapy, reirradiation, locoregional failure, treatment toxicity, immunotherapy

## Abstract

**Background/Objectives:** Locoregionally recurrent or persistent head and neck cancer (rpHNC) after prior radiotherapy carries a poor prognosis, and optimal salvage strategies remain uncertain. We evaluated survival, patterns of failure, and 90-day postoperative complications after salvage surgery combined with intraoperative electron radiation therapy (IOERT). **Methods:** We retrospectively identified 127 previously irradiated patients treated with salvage surgery and IOERT between 1999 and 2023. Overall survival (OS) and disease-free survival (DFS) were estimated using the Kaplan–Meier method, and locoregional failure (LRF) and distant failure were estimated using Aalen–Johansen cumulative incidence with death as a competing event. Associations were evaluated one predictor at a time in age- and sex-adjusted Cox (OS, DFS) and Fine-Gray (LRF) models without multiplicity adjustment, and they are exploratory. **Results:** With a median follow-up of 70 months, median overall survival (OS) was 20.5 months (95% CI, 14.6–40.2), with 1-, 2-, and 3-year OS rates of 63%, 48%, and 43%, respectively. Median disease-free survival (DFS) was 8.8 months. Positive surgical margins and extranodal extension (ENE) were associated with worse OS in separate age- and sex-adjusted models. The 1-, 2-, and 3-year cumulative incidences of LRF were 34.3%, 44.7%, and 49.8%, respectively. The corresponding cumulative incidences of distant failure were 21.6%, 24.0%, and 26.7%, respectively. Thirty-seven patients had a documented distant failure, of whom 27 (73%) experienced distant failure within one year. Available records documented three deaths within 90 days (3/127, 2.4%). **Conclusions:** Among rpHNC patients treated with this IOERT-containing multimodality salvage strategy, median OS was 20.5 months. Because the study lacked a salvage-surgery-only comparator, postoperative treatments were selectively administered, and complication reporting was incomplete, neither the efficacy nor the comparative safety of IOERT can be determined from these data.

## Simple Summary

Head and neck cancer that recurs after previous radiation is difficult to treat because further radiation may injure nearby vital tissues. We reviewed 127 patients treated at one institution with surgery and intraoperative electron radiation therapy, which delivers radiation directly to the highest-risk area during surgery while nearby tissues are moved or shielded. We assessed survival, cancer control, documented complications within 90 days, and factors associated with outcomes. Some patients achieved long-term survival, whereas cancer remaining at the surgical edge and cancer extending outside involved lymph nodes were associated with poorer outcomes. Treatment practices changed during the 24-year study, and many patients also received external radiation or drug therapy; therefore, the effect of intraoperative electron radiation therapy alone cannot be determined. Complication documentation was incomplete; therefore, the recorded events do not provide a complete estimate of treatment safety. This large, long-term experience may help clinicians select patients, refine combined salvage treatment, and design future studies using standardized treatments and complete assessment of late complications.

## 1. Introduction

Locoregionally recurrent or persistent head and neck cancer (rpHNC) following definitive or adjuvant treatment is one of the most challenging clinical scenarios in head and neck oncology. Early studies of definitive reirradiation (reRT), delivered with or without concurrent chemotherapy, reported poor long-term outcomes. Five-year OS and locoregional control were 17% and 27%, respectively, in one recurrent-disease series [1], and 14.6% and 20% in another study [2]. A larger series reported OS of 21% at two years and 9% at five years [3]. Given the challenges associated with reirradiation, salvage surgery emerged as the preferred management strategy for selected patients with resectable disease. Nevertheless, outcomes with surgery alone remained modest, with reported 5-year tumor-free actuarial survival rates of approximately 35%, reflecting a persistent risk of local and regional failure (LRF) despite complete resection [4].

These observations reinforced the need for additional local therapy to improve disease control following salvage surgery. Retrospective data suggest that postoperative reirradiation can improve locoregional control after salvage surgery [5]. A more recent multi-institutional study has reported the importance of careful patient selection for reirradiation [6]. Intraoperative electron radiation therapy (IOERT) is a technique that has been frequently implemented in rpHNC. By delivering a single, highly conformal radiation dose directly to the surgical bed at the time of resection, IOERT allows for dose intensification to high-risk areas while minimizing exposure to surrounding normal tissues [7,8,9]. Systematic reviews have emphasized that the head and neck intraoperative radiation therapy evidence remains heterogeneous and consists predominantly of retrospective institutional series [10,11,12]. Several institutional series have reported oncologic and postoperative outcomes after IOERT-containing treatment for head and neck cancer [13,14,15,16,17,18,19].

Despite these reports, the independent contribution of IOERT within multimodality salvage treatment remains uncertain, and long-term outcomes, patterns of failure, clinicopathologic associations, and postoperative complications remain incompletely characterized. Accordingly, this study aimed to characterize long-term survival and disease-control outcomes, patterns and timing of failure, clinicopathologic associations, and documented 90-day postoperative complications among previously irradiated patients treated with an IOERT-containing salvage strategy.

## 2. Materials and Methods

### 2.1. Patient Selection and Data Collection

Between 1999 and 2023, we retrospectively identified 127 patients with rpHNC who underwent salvage surgery combined with intraoperative electron radiation therapy (IOERT). All patients had previously received definitive or postoperative EBRT as part of their initial management. Persistent disease was defined as residual disease identified within six months after completion of the initial radiotherapy course, without subsequent documented disease clearance before salvage. The six-month criterion referred to initial identification of residual disease rather than the timing of salvage surgery or IOERT. Recurrent disease was defined as relapse following documented disease clearance. These definitions were applied whether the initial radiotherapy course was definitive or postoperative. The interval from recognition of persistent disease to salvage was not collected as a separate standardized variable. Distant metastatic disease associated with rpHNC was an exclusion criterion. Salvage surgery was determined by the treating head and neck multidisciplinary team based on resectability, expected morbidity, prior therapy, patient fitness, and the availability of alternative salvage approaches. Formal tumor-board documentation was not consistently available for earlier cases. Vascularized flap reconstruction was not a prespecified eligibility criterion; nevertheless, all patients underwent vascularized reconstruction, reflecting selection of patients considered fit for major salvage surgery and reconstruction.

Clinical data were collected through detailed chart review and included patient demographics (age and sex), initial disease presentation, primary tumor site, site of recurrence, and preoperative and postoperative imaging findings. Surgical details included the extent of resection and margin status. Final pathologic margins were documented as negative, close but microscopically negative, or microscopically positive. Close margins were defined as microscopically negative margins with a tumor-to-margin distance of <5 mm. For the primary binary analyses, close but microscopically negative margins were grouped with negative margins. No gross residual (R2) resections were included. Treatment-related variables included IOERT details, prescribed dose, isodose line, electron energy, and applicator cone diameter, as well as the use of post-salvage EBRT, including dose and fractionation when applicable.

Post-salvage EBRT, with or without concurrent chemotherapy, was selected on an individualized basis by the treating multidisciplinary team. Considerations generally included final pathologic risk, including margin status and ENE; the extent and anatomic location of disease; prior radiotherapy dose and anticipated field overlap; interval from prior radiotherapy; expected normal-tissue tolerance; postoperative recovery and flap status; and overall fitness. These considerations were not captured as standardized patient-level fields, particularly for earlier cases; therefore, the reason for receipt or omission of post-salvage EBRT could not be reconstructed reliably for every patient. In the analytic dataset, final margin status and ENE distributions were similar between patients who did and did not receive post-salvage EBRT; therefore, postoperative treatment comparisons were considered nonrandomized and potentially affected by residual confounding. Post-salvage EBRT, chemotherapy, and immune checkpoint inhibitors (ICI) were initiated after the index IOERT procedure and were administered nonrandomly according to clinical factors, treatment eligibility, disease course, and evolving practice patterns. These treatments were therefore summarized descriptively and were not interpreted as baseline prognostic factors or treatment-effect estimates. Cohort assembly and endpoint-specific analytic denominators are summarized in Supplementary Figure S1.

### 2.2. IOERT Parameters and Follow-Up

Among 124 patients (97.6%) with available dose data, the median IOERT dose was 12.5 Gy (IQR, 10–15 Gy; range, 7.5–17.5 Gy). Treatments were delivered en face using a mobile Mobetron electron linear accelerator (IntraOp Medical Corporation, Fremont, CA, USA). The source-to-surface distance (SSD) was fixed at 50 cm, and the applicator was positioned in direct contact with the target surface without an air gap. The median applicator cone diameter was 6 cm (IQR, 5–7 cm; range, 3–10 cm). Electron energy, applicator diameter, and bevel angle were individualized using institution-specific commissioned Mobetron depth-dose and applicator-selection tables. Prescribed monitor units were calculated using output factors corresponding to the selected energy and applicator size at 50 cm SSD. This approach is consistent with published guidance and commissioning and workflow reports for mobile IOERT systems [20,21,22,23,24]. For a 6 cm applicator with a 0° bevel, published Mobetron data report R90 depths of approximately 1.7, 2.8, and 3.8 cm for 6-, 9-, and 12-MeV electrons, respectively [25]. These values represent flat-cone reference conditions only; treatment depth and lateral isodose coverage vary with applicator diameter, bevel angle, tissue heterogeneity, and operative-bed geometry [23,25].

Before IOERT delivery, the depth and geometry of the target were assessed intraoperatively by the treating surgeon and radiation oncologist based on direct inspection and palpation of the operative bed, operative findings, and available preoperative imaging. Applicator diameter and bevel were individualized to the target geometry while limiting irradiation of adjacent normal tissues. When feasible, uninvolved critical structures, including the carotid artery, mandible, and brachial plexus, were displaced or retracted outside the applicator field or protected with internal shielding. However, when tumor directly involved or could not be separated from a critical structure, complete exclusion of that structure was not always possible without compromising target coverage. Structure-specific dosimetry was not available for this historical cohort. Prior EBRT and IOERT prescription doses were not arithmetically summed because differences in fractionation, field overlap, tissue recovery, and the dose delivered to individual anatomical structures could not be reconstructed reliably. Although follow-up examination and imaging were inconsistent over the study period, surveillance generally included clinical examination with nasopharyngoscopy and cross-sectional imaging using computed tomography (CT), magnetic resonance imaging (MRI), and/or positron emission tomography/computed tomography (PET/CT), at intervals determined by the treating team. Imaging was performed on multiple clinical systems over the study period; scanner manufacturers and models were not consistently retrievable.

### 2.3. Outcomes and Statistical Analysis

Time-to-event outcomes included DFS and OS. OS was defined as the time from IOERT to death from any cause. DFS was defined as the time from IOERT to the occurrence of disease recurrence at any site or death from any cause. Patients without documented events were censored at the date of last follow-up.

Locoregional failure (LRF) was defined as the first subsequent documented clinical, radiographic, or pathologic progression or recurrence involving the primary tumor site, surgical bed, or regional head and neck nodal basins. Microscopic margin positivity at the index resection was not counted as an LRF event; a subsequent documented progression or recurrence was required. Regional nodal failures were included regardless of their relationship to the IOERT field, although reliable in-field versus out-of-field classification was unavailable. Distant failure was defined as the first documented distant metastasis. For both failure endpoints, death without the failure of interest was treated as a competing event, and patients alive without that event were censored at their last disease assessment. All patients were retained in both cumulative-incidence analyses. Patients without a retrievable distant-disease assessment were entered as competing deaths at the date of death. Patient demographics and clinical characteristics are reported using descriptive summary statistics. OS and DFS were estimated using the Kaplan–Meier method, and median follow-up was estimated using the reverse Kaplan–Meier method. LRF and distant failure were estimated using Aalen–Johansen cumulative incidence functions.

Each predictor was evaluated separately in models adjusted for age and sex: Cox models for OS and DFS, and Fine–Gray models for LRF. Cause-specific Cox models were used as sensitivity analyses. Proportionality was assessed using scaled Schoenfeld residuals for the Cox models and covariate-by-log-time interactions for the Fine–Gray models. Because no adjustment was made for multiple comparisons, these analyses were considered exploratory and interpreted primarily through effect estimates and confidence intervals. For models with evidence of nonproportional hazards (diagnostic *p* < 0.05), exploratory hazard ratios were estimated separately for the first 12 months and after 12 months; these post hoc estimates are reported in Supplementary Table S3.

Documented postoperative complications occurring within 90 days were identified retrospectively from the available clinical records and categorized post hoc using CTCAE version 5.0 when the recorded information permitted grading. The analytic complication field contained a positive or negative statement for 75 of 127 patients and was blank or recorded as not available for 52 patients. Complication reporting was neither prospective nor standardized, and absence of documentation was not considered evidence that an event did not occur. Flap outcomes, wound complications, return to the operating room, length of stay, readmission, and late toxicity were not systematically captured. Attribution to surgery, prior radiotherapy, IOERT, post-salvage EBRT, or systemic therapy could not always be determined. Accordingly, the reported events are presented as documented complications rather than comprehensive incidence or safety estimates.

Post-salvage EBRT, chemotherapy, and ICI were given after the index IOERT procedure. Because these are post-baseline exposures, and because reliable treatment-initiation dates were unavailable for ICI, they were summarized descriptively and were not entered as baseline predictors in any survival model.

Statistical analyses were performed using R (version 4.6.1; R Foundation for Statistical Computing, Vienna, Austria). Kaplan–Meier estimates, Cox proportional-hazards models, and Schoenfeld residual tests used the survival package (version 3.8-12). Aalen–Johansen cumulative incidence estimates, Gray’s test, and Fine–Gray subdistribution-hazards models used the cmprsk package (version 2.2-12) through the tidycmprsk interface (version 1.1.2). Tables and figures used gtsummary (version 2.6.1), ggsurvfit (version 1.2.1), and ggplot2 (version 4.0.3).

## 3. Results

### 3.1. Patient Characteristics

Median follow-up was 70 months (reverse Kaplan–Meier 95% CI: 60, 113 months), and the median age was 62 years (IQR: 54, 72); 70% of patients were male, as shown in Table 1. Fifty-seven patients (45%) were treated for recurrent disease, while 70 patients (55%) had persistent disease following prior radiotherapy. The most frequent IOERT treatment sites were the neck, oropharynx, and oral cavity (23%, 19%, 18%, respectively), and the most common initial primary tumor sites were the oral cavity, oropharynx, and larynx/hypopharynx (26%, 24%, and 20%, respectively). Most tumors were SCC (86%). p16 status was available for 72 of 127 patients (57%); 27 of whom were p16-positive (38% of tested patients and 21% of the full cohort). Final pathologic margins were negative in 45 patients (35%), close but microscopically negative in 22 (17%), and microscopically positive (R1) in 60 (47%); no R2 resections were recorded. ENE was present in 14 of 58 patients with evaluable nodal disease (24%). PNI was present in 67 of 95 evaluable patients (71%), and LVI was present in 27 of 75 evaluable patients (36%).

### 3.2. Treatment Details

All 127 patients (100%) underwent reconstruction with a vascularized flap. Flap type was not consistently captured in the analytic dataset and could not be reported reliably. All patients received a first course of EBRT for the initial head and neck cancer, with a median dose of 66 Gy (IQR: 60, 70 Gy), as shown in Table 2. The median interval between the first EBRT course and IOERT was 23 months (IQR: 8, 73 months).

IOERT technical data were available for 124 of 127 patients (97.6%). The median IOERT dose was 12.5 Gy (IQR, 10–15 Gy; range, 7.5–17.5 Gy). Among these 124 patients, 119 (96.0%) were treated with 6 MeV, four (3.2%) with 9 MeV, and one (0.8%) with 12 MeV. The prescription was to the 90% IDL in 113 patients (91.1%) and to other IDLs in 11 (8.9%; range, 87–100%). The median applicator diameter was 6 cm (IQR, 5–7 cm; range, 3–10 cm).

Fifty-one patients (40%) received a second course of EBRT following salvage surgery and IOERT, and 11 patients (8.7%) received concurrent chemotherapy with the EBRT. The median second-course EBRT dose was 44 Gy (IQR: 39.6, 50 Gy), reflecting constraints of previously irradiated fields.

Twenty-four patients (19%) received ICI (16 pembrolizumab and 8 Nivolumab) at some point after salvage surgery and IOERT. The median number of completed cycles was 4.5 (IQR, 3–10). Treatment timing and indication were heterogeneous, and some patients received ICI only after subsequent disease progression; therefore, ICI exposure was summarized descriptively and was not analyzed as a baseline survival predictor.

### 3.3. Overall Survival

**Figure 1:**
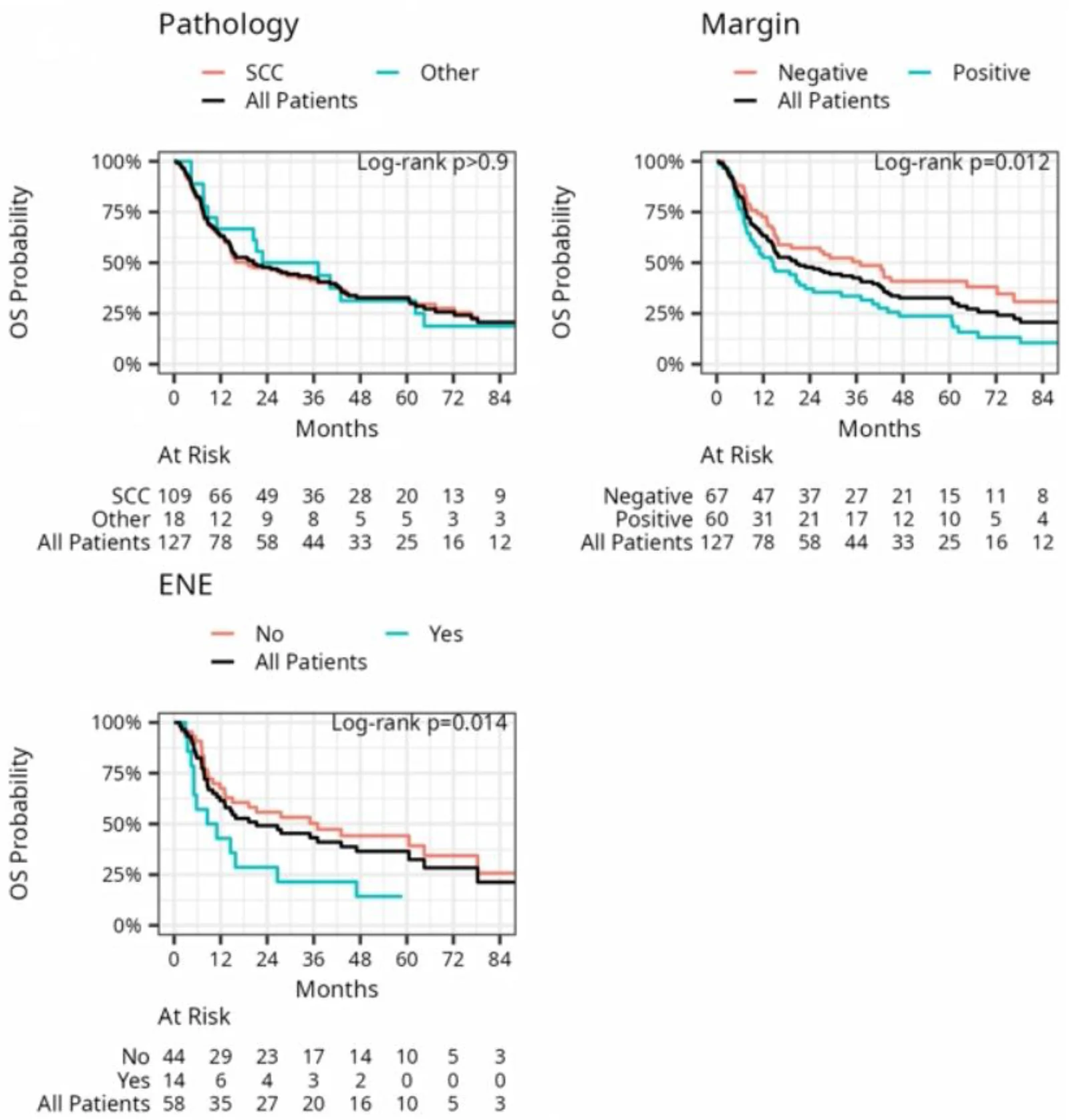
Kaplan–Meier estimates of overall survival (OS) for the study cohort. Survival curves are shown stratified by relevant clinicopathologic factors. *p*-values displayed on the figure are derived from unadjusted log-rank tests. Hazard ratios reported in the text and tables are from separate age- and sex-adjusted Cox models and represent different analyses.

At time of analysis, 35 patients (28%) were alive. Median OS was 20.5 months (95% CI, 14.6–40.2), and estimated 1-, 2-, and 3-year OS was 63%, 48%, and 43%, respectively (Table 3 and Figure 1).

In separate age- and sex-adjusted Cox models (Table 4), positive surgical margins (HR, 1.68; 95% CI 1.11–2.53; *p* = 0.014) and ENE (HR, 2.98; 95% confidence interval, 1.39–6.37; *p* = 0.007) were associated with worse OS, whereas p16 positivity was associated with better OS (HR, 0.51; 95% CI 0.27–0.97; *p* = 0.033). These associations should be considered exploratory because p16 status was unavailable in 55 patients, the analysis included mixed primary sites and histologies rather than being restricted to oropharyngeal SCC, and multiple comparisons were performed without multiplicity adjustment. These associations were obtained from separate minimally adjusted exploratory models and should not be interpreted as mutually independent prognostic effects.

### 3.4. Disease-Free Survival

**Figure 2:**
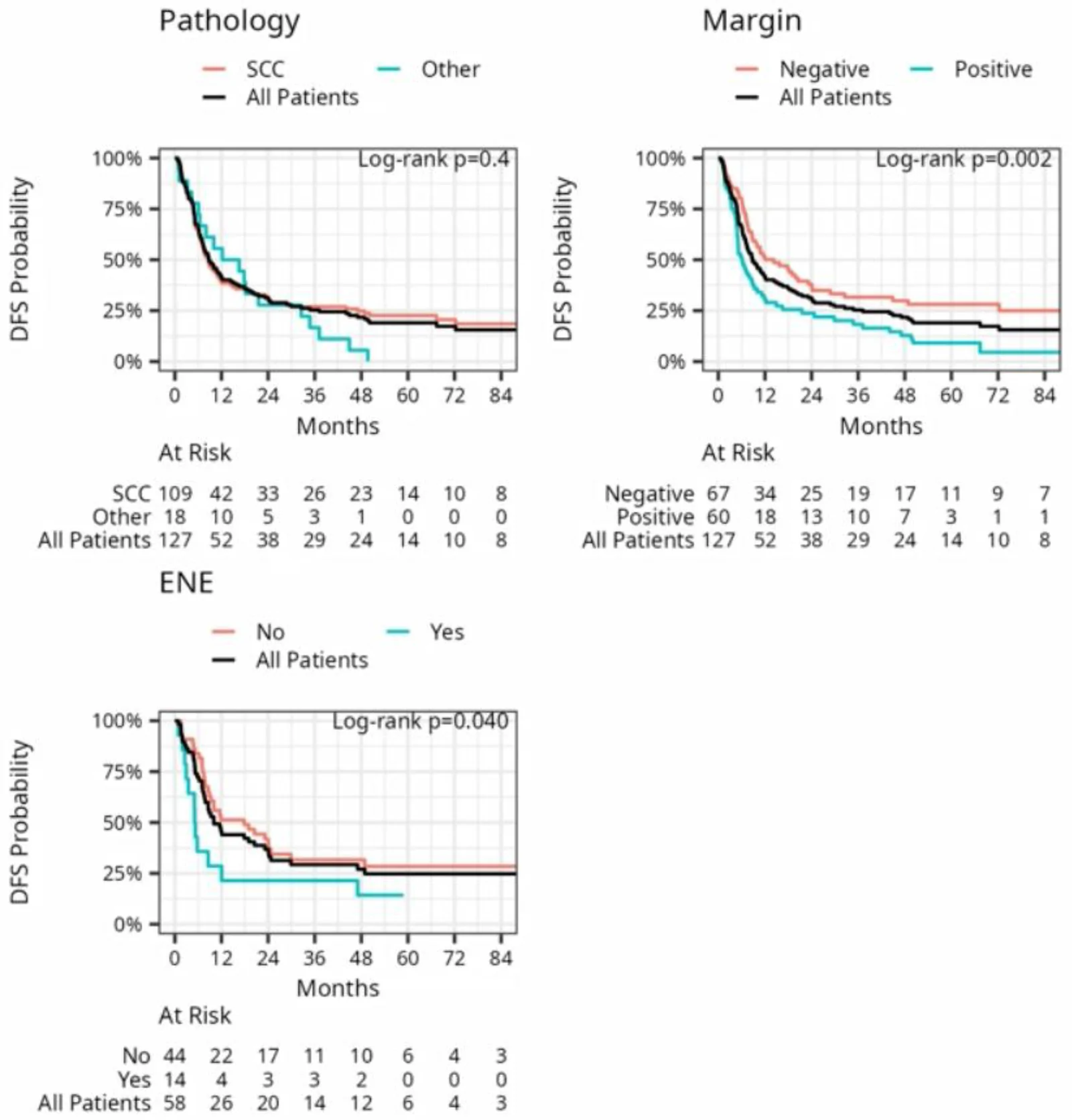
Kaplan–Meier disease-free survival plots stratified by pathology, surgical margin status, and extranodal extension. *p*-values displayed on the figure are derived from unadjusted log-rank tests. Hazard ratios reported in the text and tables are from separate age- and sex-adjusted Cox models and represent different analyses.

The median DFS was 8.8 months (95% CI: 7.2, 12.2 months); the 1-year and 3-year DFS rates were 42% and 25%, respectively (Figure 2). In separate age- and sex-adjusted exploratory Cox models, microscopically positive final pathologic margins (HR, 1.84; 95% CI, 1.24–2.72; *p* = 0.003), ENE (HR, 2.75; 95% CI, 1.32–5.74; *p* = 0.010), and LVI (HR, 1.83; 95% CI, 1.08–3.12; *p* = 0.029) were associated with shorter DFS. Complete estimates are provided in Supplementary Table S1.

### 3.5. Locoregional Failure

**Figure 3:**
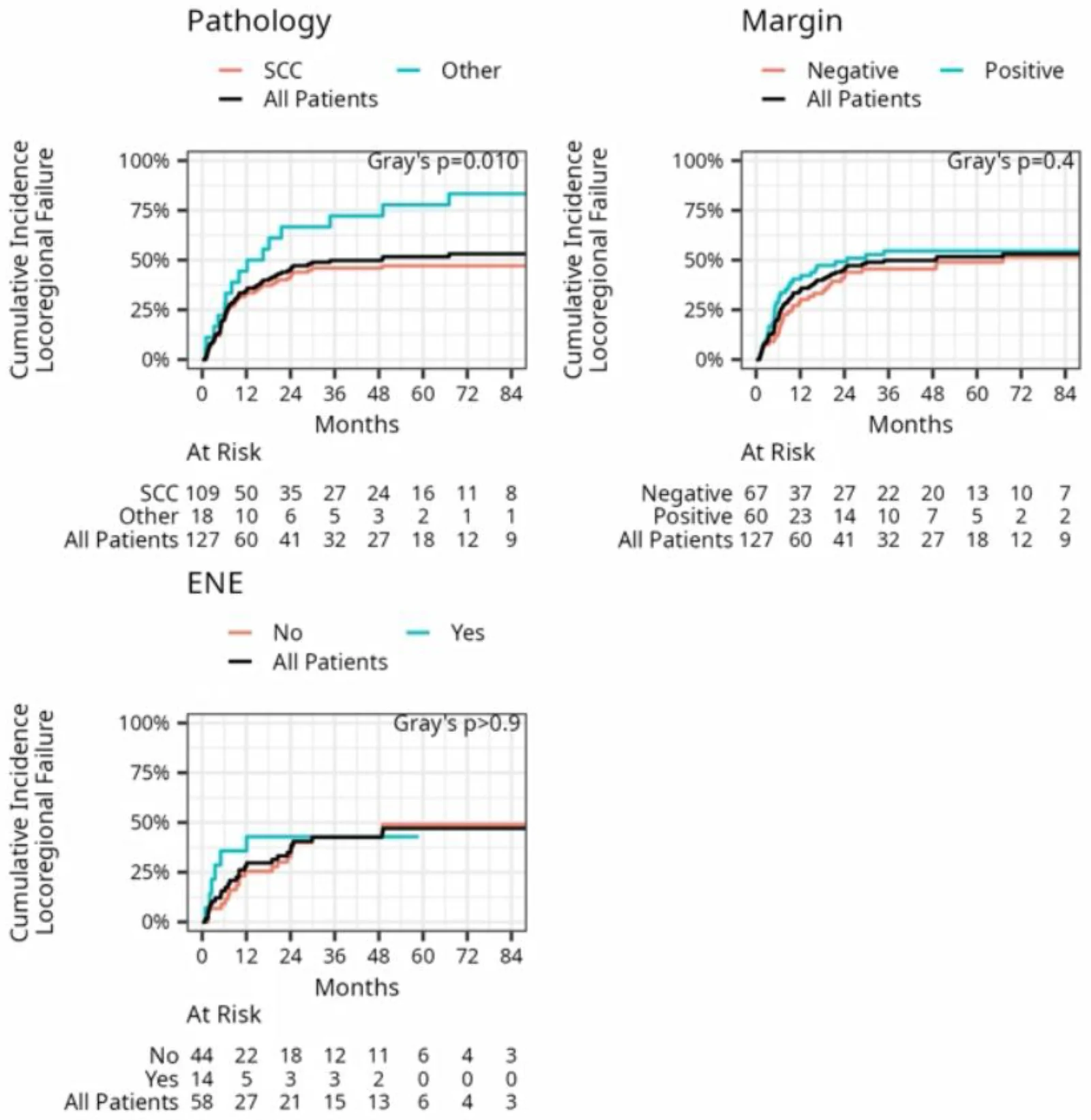
Cumulative incidence of locoregional failure, with death without prior locoregional failure treated as a competing event. *p*-values displayed on the figure are from unadjusted Gray’s tests. Subdistribution hazard ratios are from separate age- and sex-adjusted Fine–Gray models.

Sixty-five of 127 patients experienced LRF: 21 (16.5%) had local failure alone, 11 (8.7%) had regional failure alone, and 33 (26.0%) had both local and regional failure. With death without prior LRF treated as a competing event, the 1-, 2-, and 3-year cumulative incidences of LRF were 34.3% (95% CI, 26.1–42.6), 44.7% (95% CI, 35.8–53.3), and 49.8% (95% CI, 40.7–58.3), respectively (Figure 3). In a separate age- and sex-adjusted Fine–Gray model, PNI was not statistically associated with LRF (sHR, 2.03; 95% CI, 0.98–4.19; *p* = 0.056). This borderline association should be interpreted as exploratory because multiple comparisons were performed without multiplicity adjustment. The three-year cumulative incidence of LRF was 72% among the 18 patients with non-SCC histology and 46% among the 109 patients with SCC (unadjusted Gray’s *p* = 0.01). In the age- and sex-adjusted Fine–Gray model, the corresponding association was nonsignificant (sHR, 1.78; 95% CI, 0.98–3.22; *p* = 0.058). Because the non-SCC group was small and included several biologically distinct histologies, this result is interpreted as exploratory. Fine–Gray and cause-specific Cox estimates for all evaluated predictors are provided in Supplementary Table S2.

### 3.6. Distant Failure

Post-IOERT distant failure assessment was retrievable for 115 patients, and data were not available for 12 patients, all of whom died without a recorded distant-failure date. These patients were treated before 2011 and had a median OS of 7.1 months. All 127 patients were included in the cumulative-incidence analysis. Thirty-seven patients had a documented distant failure, and 27 of them (73%) occurred within one year. The median observed time from IOERT to distant failure was 5.3 months (IQR, 3.7–14.3). The 1-, 2-, and 3-year cumulative incidences of distant failure were 21.6% (95% CI, 14.8–29.1), 24.0% (95% CI, 16.9–31.8), and 26.7% (95% CI, 19.2–34.8), respectively.

### 3.7. Ninety-Day Postoperative Complications

Vital-status data were available for all 127 patients. All-cause mortality was 1/127 (0.8%) within 30 days and 3/127 (2.4%) within 90 days of salvage surgery and IOERT.

The complication field contained a documented positive or negative statement for 75 patients and was unavailable for 52. Among the 75 patients with complication documentation, available records described mucocutaneous toxicity in six (8.0%; 6/127, 4.7% of the full cohort), dysphagia in four (5.3%; 4/127, 3.1%), and wound dehiscence with carotid artery dissection in one (1.3%; 1/127, 0.8%). Four patients had fistula (5.3%; 4/127, 3.1%); one occurred on day 91, whereas the timing of the other three was not recorded consistently. Because the timing and grade of individual events were incompletely documented, an overall grade 3–4 complication rate could not be determined reliably.

The fatal carotid rupture was classified as a grade 5 event and occurred in a different patient from the patient with carotid artery dissection. The rupture occurred 18 days after salvage surgery in a patient with extensive carotid encasement and prior EBRT, despite vascularized flap coverage. The other two deaths within 90 days were attributed to sepsis. These findings represent documented events rather than a comprehensive estimate of postoperative morbidity.

## 4. Discussion

To our knowledge, this is the largest reported cohort restricted to previously irradiated patients treated with salvage surgery and IOERT for rpHNC. Wald et al. previously reported 61 patients treated at this institution between 2006 and 2014, of whom 58 had received prior EBRT [16]. After direct patient-identifier matching, all previously irradiated patients were included in the present cohort. The current study adds 69 previously unreported patients, extends accrual through 2023, and reports a median follow-up of 70 months using the reverse Kaplan–Meier method.

We observed a median OS of 20.5 months, with 1-, 2-, and 3-year OS rates of 63%, 48%, and 43%, respectively. Despite intensive local treatment with salvage surgery and IOERT, DFS remained limited (median, 8.8 months), reflecting the high-risk nature of this population and the frequency of early disease failure. These outcomes describe an IOERT-containing multimodality salvage strategy rather than the isolated effect of IOERT. After accounting for death as a competing event, the three-year cumulative incidence of LRF was 49.8%. The combined locoregional endpoint included both local and regional nodal failure, but reliable classification according to failure within or outside the IOERT field was unavailable. The higher cumulative incidence of LRF observed in the non-SCC group should be interpreted cautiously. This group included only 18 patients and comprised several biologically distinct tumor types. The finding may reflect differences in tumor biology, case selection, or competing mortality, but the small subtype counts do not permit histology-specific conclusions.

Early experiences with surgery and IOERT for rpHNC demonstrated that outcomes were influenced by different variables. Garrett et al. reported outcomes after surgery and IOERT in 28 patients, with an estimated 1-year OS of 67%. Local failure rates were 13%, 25%, and 100% among patients with close, microscopically positive, and gross residual disease (R2), respectively. In our cohort, 22 margins were recorded as close (<5 mm) but microscopically negative and were grouped with negative margins in the primary analysis. Therefore, our binary comparison cannot be directly equated with the three categories reported by Garrett et al. They also identified carotid blowout as the major treatment-related complication, occurring predominantly when tumor abutted or invaded the carotid artery [26].

Similarly, Rate et al. analyzed 47 previously irradiated patients treated with salvage surgery and IOERT to a median dose of 20 Gy, reporting 2-year OS of 55% and LRC of 62%. Outcomes again favored patients with microscopic rather than gross residual disease, with a trend toward improved OS and LC [27].

Subsequent series highlighted the limitations of IOERT when used without effective systemic therapy or postoperative EBRT. Nag et al. reported a single-institution experience in 38 previously irradiated patients who had salvage maximal resection and IOERT (median 15 Gy) without postoperative EBRT. Locoregional failure (LRF) was frequent and early, with 1- and 2-year LRC rates of 11% and 4%, respectively, and a median OS of 7 months, underscoring that salvage surgery plus IOERT alone was insufficient for durable disease control in these patients [13]. Interestingly, Schleicher et al. evaluated IOERT alone (without salvage surgery) in a predominantly palliative context in 84 previously irradiated patients, achieving symptom palliation in most cases but with a median OS of only 6.8 months and rapid local progression, further emphasizing that IOERT without curative-intent resection or systemic disease control cannot achieve long-term benefit [14]. Similarly, Chen et al. reported outcomes in 137 patients treated with gross resection and IORT; 113 had received prior EBRT, and 94 had SCC. The reported 3-year LRC and OS rates were 51% and 36%, respectively [15], broadly consistent with the present study (3-year OS 43%).

In the absence of prospective randomized trials evaluating IOERT-based approaches, salvage surgery followed by adjuvant chemoradiation has remained the standard of care, largely based on the randomized trial by Janot and colleagues. In that study, postoperative chemoradiation improved DFS without a statistically significant improvement in 3-year OS (approximately 30–35%, *p* = 0.50), at the cost of substantial acute and late toxicity [28].

Post-salvage EBRT and chemotherapy were administered nonrandomly after the index IOERT procedure. Although measured margin and ENE distributions were similar between the postoperative EBRT groups, treatment selection may have depended on additional clinical factors that were incompletely captured. Comparisons according to postoperative treatment receipt therefore cannot determine whether these therapies were beneficial, ineffective, or harmful.

Janot et al. prospectively collected acute and late toxicity in a randomized trial [28], whereas the present study relied on retrospective documentation of events within 90 days; therefore, the results are not directly comparable. Three deaths were documented within 90 days of salvage surgery and IOERT (3/127, 2.4%).

Because surgical complications, flap outcomes, readmissions, and late toxicities were not systematically reported, the present data cannot establish the comparative safety of IOERT or determine the contribution of IOERT to individual complications. The carotid artery rupture occurred after salvage surgery in a patient with extensive tumor encasement of more than 180° of the carotid artery and prior EBRT, despite vascularized flap coverage. The contributions of tumor anatomy, prior radiation injury, surgery, and IOERT cannot be determined. Structure-specific dosimetry was not available for this patient or the other historical severe-toxicity cases; therefore, cumulative doses to the implicated anatomical structures could not be estimated reliably.

Early distant failure was common: 73% of distant metastases occurred within the first year. This pattern suggests that occult systemic disease may have been present in some patients at salvage and supports prospective evaluation of systemic therapy. Immune checkpoint inhibitors (ICI) improve survival in recurrent or metastatic head and neck squamous cell carcinoma [29], and a phase II trial of adjuvant nivolumab after salvage resection reported encouraging DFS with acceptable tolerability [30]. The HNSALV phase I trial is evaluating pembrolizumab with salvage surgery and IOERT for persistent or recurrent disease (NCT04754321). High-dose focal radiation may promote immunogenic cell death, tumor-antigen release, innate immune signaling, and T-cell priming, providing a biological rationale for combination with immune checkpoint blockade. However, these effects depend on radiation dose, fractionation, timing, treated volume, and the tumor microenvironment, and any interaction between IOERT and ICI remains hypothetical in this setting [31].

In the present study, ICI exposure was heterogeneous in timing and indication, and some patients initiated treatment only after subsequent disease progression. Patients selected to receive ICI may also have differed in performance status, age, renal or hepatic function, disease burden, recurrence status, and general treatment eligibility. Because ICI receipt and completed-cycle count were post-baseline exposures and reliable treatment-start dates were unavailable, the present analysis cannot estimate an ICI treatment effect or an interaction between ICI and IOERT. Therefore, the receipt of ICI is reported descriptively only, and potential combinations of IOERT and ICI require prospective evaluation.

### Strengths and Limitations

Strengths include cohort size, long follow-up, technical description of IOERT delivery, and characterization of failure timing in a difficult salvage population. The observation that distant failures clustered early provides clinically relevant context for future multimodality studies.

The principal limitations are the retrospective design, absence of a salvage-surgery-only control arm, and the 24-year accrual period. Calendar era represents a potential confounder because imaging, staging, reconstructive surgery, EBRT planning and delivery, supportive care, systemic therapy, and patient-selection practices changed substantially between 1999 and 2023. Detailed first-course radiotherapy technique, previous systemic regimens, previous surgery, and formal tumor-board documentation were not consistently available, particularly for earlier cases and externally treated patients. All patients underwent vascularized flap reconstruction, reflecting a fit and highly selected surgical population and limiting generalizability to patients unable to undergo major salvage resection and reconstruction. Disease status was reported descriptively because the interval between recognition of persistent disease and salvage treatment was not captured uniformly.

Locoregional failures could be classified as local or regional but not reliably as within or outside the IOERT field. In patients with microscopically positive margins, the available retrospective data also could not distinguish progression of persistent microscopic disease from recurrence following documented disease clearance. Distant-failure data were unavailable in 12 predominantly early-era patients who died without a recorded distant-failure date; therefore, the reported cumulative incidence reflects documented distant failure and may underestimate clinically occult metastatic disease.

Post-salvage EBRT, chemotherapy, and ICI were administered after the index IOERT procedure and were influenced by clinical selection, treatment eligibility, calendar era, and subsequent disease course. These treatments were therefore not analyzed as baseline prognostic or treatment-effect variables. Their benefit or lack of benefit cannot be determined from the present nonrandomized study. The cohort also included 18 patients with non-SCC histology, and p16 analyses were not restricted to oropharyngeal squamous cell carcinoma.

The clinicopathologic associations were derived from separate age- and sex-adjusted exploratory models and should not be interpreted as independent effects. Complete-case analysis was used for variables with missing data. Several models did not satisfy the applicable proportionality assumption. Their overall hazard ratios should therefore be interpreted as time-averaged associations; exploratory 12-month time-split estimates are provided in Supplementary Table S3. Complication ascertainment was retrospective and incomplete, with the complication field blank or unavailable for 52 of 127 patients. Consequently, the reported complication counts represent lower bounds and cannot support comparative safety conclusions.

## 5. Conclusions

Among rpHNC patients treated with this IOERT-containing multimodality salvage strategy, median OS was 20.5 months. Positive margins and ENE were associated with worse survival outcomes. Because this retrospective study lacked a salvage surgery-only control arm, postoperative treatments were selectively administered, and complication reporting was incomplete; neither the efficacy nor the comparative safety of IOERT can be determined from these data. Locoregional and distant failure were evaluated in the presence of substantial competing mortality, with three-year cumulative incidences of 49.8% and 26.7%, respectively. Early distant metastasis remained a major limitation to durable disease control. These findings support prospective studies that standardize patient selection, integrate systemic therapy when appropriate, use competing-risk and time-dependent analyses, and systematically capture late complications and cumulative radiation dose.

## Figures and Tables

**Table 1:** Patient characteristics.

Characteristic	N = 127 ^1^
**Age (years)**	62 (54, 72)
**Sex**	
Female	38 (30.0%)
Male	89 (70.0%)
**Primary tumor site**	
Oral cavity	33 (26.0%)
Oropharynx	30 (24.0%)
Larynx/hypopharynx	25 (20.0%)
Sinonasal	14 (11.0%)
Other	13 (10.0%)
Salivary gland	12 (9.4%)
**IOERT Treatment Site**	
Neck	29 (23.0%)
OP	24 (19.0%)
OC	23 (18.0%)
Larynx/Hypopharynx	14 (11.0%)
Sinonasal	13 (10.0%)
Other	12 (9.4%)
Salivary	10 (7.9%)
Nasopharynx	2 (1.6%)
**Pathology**	
SCC	109 (85.8%)
Non-SCC histology	18 (14.2%)
Adenocarcinoma	5 (3.9%)
Adenoid cystic carcinoma	5 (3.9%)
Sarcoma	4 (3.1%)
Mucoepidermoid carcinoma	2 (1.6%)
Carcinoma ex-pleomorphic adenoma	1 (0.8%)
Anaplastic thyroid carcinoma	1 (0.8%)
**Positive p16**	27/72 (38.0%)
Unknown	55
Final pathologic margin status	
Negative	45 (35.4%)
Close, microscopically negative (<5 mm)	22 (17.3%)
**Microscopically positive (R1)**	60 (47.2%)
**Positive ENE**	14/58 (24.0%)
**Positive PNI**	67/95 (71.0%)
Unknown	32
**Positive LVI**	27/75 (36.0%)
Unknown	52
**Disease status**	
Recurrent	57 (44.9%)
Persistent	70 (55.1%)

^1^ Data are presented as median (interquartile range), *n* (%), or *n*/*N* (%), where *n* is the observed count and *N* is the total cohort or the relevant denominator. Percentages are calculated among evaluable cases unless otherwise specified. OP, oropharynx; OC, oral cavity; SCC, squamous cell carcinoma; ENE, extranodal extension; PNI, perineural invasion; LVI, lymphovascular invasion. Close margins were defined as microscopically negative margins with a tumor-to-margin distance < 5 mm. Close and negative margins were combined in the primary binary outcome analyses.

**Table 2:** Treatment details.

Characteristic	N = 127 ^1^
**First Course RT Dose, Gy, n = 122**	66 (60, 70)
**Time interval between end of first RT and IOERT dates, months, n = 127**	23 (8, 73)
IOERT dose **, Gy	12.5 (IQR, 10–15; range, 7.5–17.5)
Energy (MeV) **	n = 124
6 MeV	119
9 MeV	4
12 MeV	1
Missing data	3
Isodose **	
90%	113
Other isodose levels (range: 87–100%)	11
Missing data	3
Applicator cone diameter **, cm	6 (IQR, 5–7; range, 3–10)
**Second Course RT**	51 (40%)
Post-IOERT EBRT Dose (Gy) ***	44 (39.6, 50)
**Post-salvage treatment groups**	
IOERT	76 (60%)
IOERT+EBRT	40 (31%)
IOERT+EBRT+Chemo	11 (8.7%)
**Received immune checkpoint inhibitors (ICI)**	24 (19%)
Number of ICI cycles completed †	4.5 (3, 10)

^1^ Median (Q1, Q3); n (%) ** IOERT data available for 124 patients. *** Post-IOERT EBRT dose available for 50 patients. † Reported for patients receiving immunotherapy only. Cycles refer to systemic ICI administered at any point following salvage surgery + IOERT.

**Table 3:** Cumulative incidence of locoregional failure and Kaplan–Meier estimates of disease-free and overall survival, with 95% confidence intervals.

Strata	N	LRF				DFS					OS				
		1 Year	2 Years	3 Years	Gray’s Test *p*-Value	Median, Months	1 Year	2 Years	3 Years	Log-Rank *p*-Value	Median, Months	1 Year	2 Years	3 Years	Log-Rank *p*-Value
All patients	127	34% (26%, 43%)	45% (36%, 53%)	50% (41%, 58%)		8.8 (7.2, 12.2)	42% (34%, 51%)	31% (24%, 41%)	25% (19%, 34%)		20.5 (14.6, 40.2)	63% (55%, 72%)	48% (40%, 57%)	43% (35%, 52%)	
Histology (SCC vs. non-SCC)					0.01					0.4					>0.9
non-SCC	18	44% (21%, 66%)	67% (39%, 84%)	72% (44%, 88%)		14.4 (6.4, 34.8)	56% (37%, 84%)	28% (13%, 59%)	17% (6%, 47%)		30 (11, NA)	67% (48%, 92%)	50% (32%, 79%)	50% (32%, 79%)	
SCC	109	33% (24%, 42%)	41% (32%, 50%)	46% (36%, 55%)		8.6 (7, 11.8)	39% (31%, 50%)	32% (24%, 42%)	27% (20%, 37%)		18.8 (14.3, 41.7)	63% (54%, 73%)	47% (39%, 58%)	41% (33%, 52%)	
Final pathologic margin status					0.4					0.002					0.012
Negative or close	67	29% (18%, 40%)	41% (29%, 52%)	46% (33%, 57%)		14.3 (8.8, 24.2)	52% (41%, 65%)	38% (28%, 52%)	32% (22%, 45%)		37 (15.8, 76.6)	73% (63%, 84%)	57% (46%, 71%)	50% (40%, 64%)	
Positive	60	41% (28%, 53%)	49% (36%, 61%)	55% (41%, 67%)		6.1 (5, 10)	31% (21%, 45%)	24% (15%, 38%)	18% (11%, 32%)		14.5 (8.7, 32.1)	53% (41%, 67%)	37% (27%, 52%)	34% (23%, 48%)	
ENE (regional nodal cases only)					>0.9					0.04					0.014
No	44	25% (14%, 39%)	33% (19%, 47%)	43% (27%, 57%)		17.8 (9.3, 29.9)	51% (38%, 69%)	42% (29%, 60%)	32% (20%, 50%)		37 (13.2, NA)	68% (55%, 83%)	56% (43%, 73%)	50% (37%, 68%)	
Yes	14	36% (12%, 60%)	43% (16%, 67%)	43% (16%, 67%)		5.2 (3.5, NA)	29% (12%, 65%)	21% (8%, 58%)	21% (8%, 58%)		9.8 (5.1, NA)	43% (23%, 78%)	29% (12%, 65%)	21% (8%, 58%)	

CI, confidence interval; DFS, disease-free survival; ENE, extranodal extension; LRF, locoregional failure; NA, not estimable; OS, overall survival; SCC, squamous cell carcinoma. Values are estimates (95% CI). LRF was estimated using the cumulative-incidence function, with death without prior LRF treated as a competing event. DFS and OS were estimated using the Kaplan–Meier method. *p*-values are unadjusted and are from Gray’s test for LRF and log-rank tests for DFS and OS. Close but microscopically negative margins were grouped with negative margins for these analyses.

**Table 4:** Separate age- and sex-adjusted Cox proportional hazards models for overall survival; each model included one predictor of interest plus age and sex.

Characteristic	N	Event N	HR	95% CI	*p*-Value
Pathology					0.5
SCC	109	76	—	—	
Non-SCC	18	16	0.83	0.45, 1.50	
Final pathologic margin status *					0.014
Negative or close	67	42	—	—	
Positive	60	50	1.68	1.11, 2.53	
ENE if Nodal					0.007
No	44	26	—	—	
Yes	14	12	2.98	1.39, 6.37	
PNI					0.4
No	28	19	—	—	
Yes	67	49	1.24	0.73, 2.12	
LVI					0.075
No	48	31	—	—	
Yes	27	23	1.68	0.95, 2.97	
Applicator cone diameter, per 1 cm increase **	124	90	1.05	0.92, 1.20	0.4
p16 Status ***					0.033
Negative	45	33	—	—	
Positive	27	14	0.51	0.27, 0.97	
Interval between completion of first-course RT and IOERT, continuous, per year	127	92	0.96	0.93, 1.00	0.056
Interval between completion of first-course RT and IOERT, categorical					0.10
(0,1)	45	35	—	—	
[1,4)	33	20	0.58	0.33, 1.01	
[4,∞)	49	37	0.65	0.40, 1.05	

HR, hazard ratio; CI, confidence interval. *p*-values shown on variable-header rows are global likelihood-ratio *p*-values. No adjustment for multiple comparisons was performed. Each predictor was evaluated in a separate model adjusted for age and sex. Proportional-hazards assumptions were evaluated using scaled Schoenfeld residuals. * Margin status was recorded as negative, close, or microscopically positive. For time-to-event analyses, close margins were grouped with negative margins. No gross residual (R2) resections were included. ** Applicator cone diameter was modeled per 1 cm increase and included exploratorily as an imperfect surrogate for the lateral extent of the treated operative bed. *** p16 analysis included 72 patients with available results and was not restricted to oropharyngeal SCC.

## Data Availability

The datasets generated and/or analyzed during the current study are available from the corresponding author upon reasonable request.

## Supplementary Materials

The following supporting information can be downloaded at: https://www.mdpi.com/article/10.3390/cancers18203399/s1, Figure S1: Cohort assembly and analytic denominators; Table S1: Disease-free survival—separate Cox models, one predictor per model, each adjusted for age and sex; Table S2: Locoregional failure—separate models, one predictor per model, each adjusted for age and sex; Table S3: Time-split hazard ratios for predictors that violate proportional hazards (PH test *p* < 0.05).

## Abbreviations

Abbreviation	Definition
CI	Confidence interval
CT	Computed tomography
CTCAE	Common Terminology Criteria for Adverse Events
DFS	Disease-free survival
EBRT	External-beam radiation therapy
ENE	Extranodal extension
ICI	Immune checkpoint inhibitor
IDL	Isodose line
IOERT	Intraoperative electron radiation therapy
IQR	Interquartile range
LRF	Locoregional failure
LVI	Lymphovascular invasion
MRI	Magnetic resonance imaging
OS	Overall survival
PET	Positron-emission tomography
PNI	Perineural invasion
rpHNC	Recurrent or persistent head and neck cancer
SCC	Squamous cell carcinoma
SSD	Source-to-surface distance
