# Supplementary material for: Salvage Surgery with Intraoperative Electron Radiation Therapy for Recurrent or Persistent Head and Neck Cancer After Prior Radiotherapy
Source: Cancers (Basel). 2026 Oct 21;18(20):3399. doi: 10.3390/cancers18203399 (PMC13702705; doi:10.3390/cancers18203399)
Supplement: Supplementary file 1 [file cancers-18-03399-s001.zip › cancers-4531891-supplementary.pdf]

cancers

MDPI

Supplementary Materials: Salvage Surgery with Intraoperative Electron Radiation Therapy for Recurrent or Persistent Head and Neck Cancer After Prior Radiotherapy

Ahmed N. Elguindy, Sujith Baliga, Brett G. Klamer, Allison Palmiero, Khaled N. Dibs, Andrew Koempel, Emile Gogineni, David Konieczkowski, Darrion L. Mitchell, Nolan Seim, Kyle VanKoevering, Catherine Haring, Lauren Miller, Stephen Y. Kang, Amit Agrawal, Enver Ozer, James W. Rocco, Ricardo L. Carrau, Matthew Old, Marcelo Bonomi, Priyanka Bhateja, Arnab Chakravarti, John Grecula and Dukagjin M. Blakaj

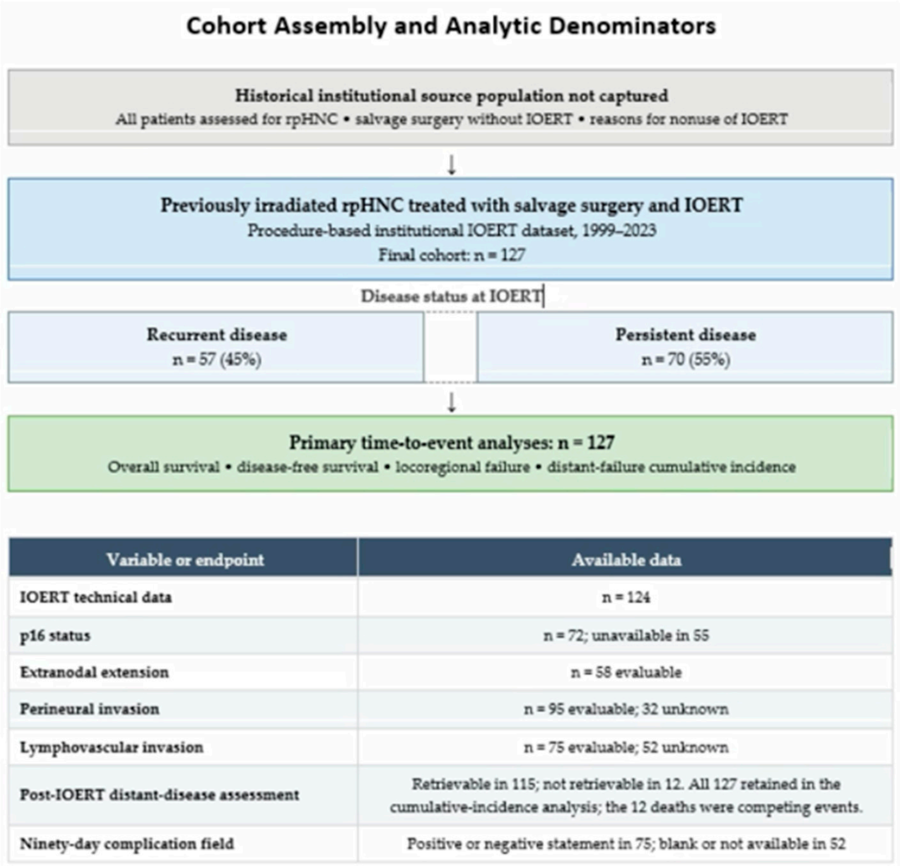

Figure S1. Cohort assembly and analytic denominators. All 127 included patients contributed to the OS, DFS, LRF, and distant-failure cumulative-incidence analyses. **Abbreviations:** DFS, disease-free survival; IOERT, intraoperative electron radiation therapy; LRF, locoregional failure; OS, overall survival; rpHNC, recurrent or persistent head and neck cancer.

Table S1. Disease-free survival. Separate Cox models, one predictor per model, each adjusted for age and sex. p-value is the global likelihood ratio test. Schoenfeld p tests a non-zero slope in the scaled Schoenfeld residuals of the predictor. No adjustment for multiple comparisons.

Characteristic	N	Event N	HR	95% CI	p-value	Schoenfeld p
Pathology					>0.9	0.015
SCC	109	85	—	—		

Characteristic	N	Event N	HR	95% CI	p-value	Schoenfeld p
Other	18	18	1.00	0.56, 1.76		
Final pathologic margin status					0.003	0.7
Negative or close	67	49	—	—		
Positive	60	54	1.84	1.24, 2.72		
ENE if nodal					0.010	0.14
No	44	30	—	—		
Yes	14	12	2.75	1.32, 5.74		
PNI					0.091	0.3
No	28	20	—	—		
Yes	67	56	1.53	0.92, 2.56		
LVI					0.029	>0.9
No	48	36	—	—		
Yes	27	24	1.83	1.08, 3.12		
Cone diameter, per cm	124	101	0.98	0.86, 1.11	0.7	0.004
p16 status					0.10	0.6
Negative	45	36	—	—		
Positive	27	18	0.62	0.35, 1.11		
Interval from first-course RT to IOERT, per year	127	103	0.98	0.94, 1.02	0.3	0.3
Interval from first-course RT to IOERT, years					0.3	0.10
(0,1)	45	38	—	—		
[1,4)	33	25	0.69	0.41, 1.14		
[4,Inf)	49	40	0.81	0.51, 1.27		

Abbreviations: CI = Confidence Interval, HR = Hazard Ratio

Table S2. Locoregional failure. Separate models, one predictor per model, each adjusted for age and sex. Fine-Gray models treat death without LRF as a competing event. Cause-specific Cox models censor death without LRF. Fine-Gray p-value is the global Wald test with sandwich variance, and time interaction p tests a predictor by log-time interaction. Cause-specific p-value is the global likelihood ratio test, and Schoenfeld p tests a non-zero slope in the scaled Schoenfeld residuals of the predictor. No adjustment for multiple comparisons.

Characteristic	Fine-Gray subdistribution hazard						Cause-specific hazard			
	N	Event N	sHR	95% CI	p-value	Time interaction p	HR	95% CI	p-value	Schoenfeld p
Pathology					0.058	0.14			0.5	0.4
SCC	109	50	—	—			—	—		
Other	18	15	1.78	0.98, 3.22			1.29	0.67, 2.49		
Final pathologic margin status					0.5	0.13			0.14	0.3
Negative or close	67	33	—	—			—	—		
Positive	60	32	1.19	0.73, 1.92			1.45	0.89, 2.37		
ENE if nodal					0.6	0.012			0.2	0.084
No	44	20	—	—			—	—		
Yes	14	6	1.30	0.44, 3.86			1.84	0.69, 4.89		
PNI					0.056	0.5			0.034	0.6
No	28	9	—	—			—	—		
Yes	67	37	2.03	0.98, 4.19			2.09	1.01, 4.34		
LVI					0.2	0.8			0.10	0.5
No	48	21	—	—			—	—		
Yes	27	16	1.60	0.81, 3.17			1.77	0.91, 3.44		
Cone diameter, per cm	124	65	0.93	0.79, 1.11	0.4	0.003	0.96	0.82, 1.13	0.6	0.005
p16 status					0.4	0.5			0.091	0.7
Negative	45	22	—	—			—	—		
Positive	27	11	0.73	0.36, 1.47			0.54	0.26, 1.12		
Interval from first-course RT to IOERT, per year	127	65	1.02	0.97, 1.07	0.5	0.4	1.00	0.95, 1.05	>0.9	0.4
Interval from first-course RT to IOERT, years					0.083	0.025			0.14	0.051
(0,1)	45	25	—	—			—	—		

https://doi.org/10.3390/xxxxx

Characteristic	Fine-Gray subdistribution hazard						Cause-specific hazard			
	N	Event N	sHR	95% CI	p-value	Time interaction p	HR	95% CI	p-value	Schoenfeld p
[1,4)	33	12	0.48	0.24, 0.96			0.51	0.25, 1.01		
[4,Inf)	49	28	0.92	0.53, 1.61			0.78	0.45, 1.36		

Abbreviations: CI = Confidence Interval, HR = Hazard Ratio

Abbreviations: CI, confidence interval; HR, hazard ratio; sHR, subdistribution hazard ratio.

Table S3. Time-split hazard ratios for the predictors that violate proportional hazards (PH test p < 0.05). Each model is adjusted for age and sex. The predictor has one hazard ratio for the first 12 months and one after 12 months. The late hazard ratio applies to patients still at risk at 12 months. Fine-Gray rows are subdistribution hazard ratios. PH test p is the Schoenfeld test for Cox models and the log-time interaction test for Fine-Gray models. p difference tests that the two hazard ratios are equal.

Endpoint	Model	Predictor	Level	PH test p	N	Events 0-12 mo	Events >12 mo	HR 0-12 mo (95% CI)	HR >12 mo (95% CI)	p difference
OS	Cox	LVI	Yes	0.022	75	27	27	0.73 (0.32, 1.68)	4.49 (1.86, 10.83)	0.003
		Cone diameter, per cm		0.003	124	44	46	1.29 (1.08, 1.55)	0.83 (0.68, 1.02)	0.001
		p16 status	Positive	0.038	72	22	25	0.28 (0.09, 0.81)	0.80 (0.36, 1.82)	0.12
		Interval from first-course RT to IOERT, per year		0.002	127	46	46	0.90 (0.82, 0.98)	0.99 (0.95, 1.03)	0.046
		Interval from first-course RT to IOERT, years	[1,4)	0.004	127	46	46	0.46 (0.22, 0.96)	0.84 (0.36, 1.99)	0.056
			[4,Inf)		127	46	46	0.36 (0.18, 0.73)	1.24 (0.60, 2.57)	
DFS	Cox	Pathology	Other	0.015	127	73	30	0.53 (0.24, 1.15)	3.30 (1.42, 7.67)	<0.001
		Cone diameter, per cm		0.004	124	71	30	1.10 (0.95, 1.27)	0.69 (0.53, 0.90)	0.002
LRF	Cause-specific Cox	Cone diameter, per cm		0.005	124	43	22	1.10 (0.91, 1.32)	0.70 (0.51, 0.96)	0.017
	Fine-Gray	ENE if nodal	Yes	0.012	58	16	10	2.05 (0.64, 6.56)	0.45 (0.05, 4.07)	0.2
		Cone diameter, per cm		0.003	124	43	22	1.07 (0.89, 1.30)	0.64 (0.44, 0.94)	0.023
		Interval from first-course RT to IOERT, years	[1,4)	0.025	127	43	22	0.34 (0.15, 0.76)	1.50 (0.36, 6.18)	0.017
			[4,Inf)		127	43	22	0.52 (0.27, 1.02)	3.97 (1.16, 13.58)	
